# One-Step Anodic Synthesis of Gd-Doped TiO_2_ Nanotubes for Enhanced Photocatalysis

**DOI:** 10.3390/ma19030610

**Published:** 2026-02-04

**Authors:** Xing Lv, Zhixiong Xie, Maodong Kang, Shijie Dong

**Affiliations:** 1 Hubei Key Laboratory of Green Light Industry Materials, Hubei Engineering Laboratory of Automotive Lightweight Materials and Processing, School of Materials and Chemical Engineering, Hubei University of Technology, Wuhan 430068, China; 13297223688@163.com (X.L.); dongsjsj@163.com (S.D.); 2 Shanghai Key Laboratory of Advanced High-Temperature Materials and Precision Forming and State Key Laboratory of Metal Matrix Composites, School of Materials Science and Engineering, Shanghai Jiao Tong University, Shanghai 200240, China; kangmd518@sjtu.edu.cn

**Keywords:** Gd-doped TiO_2_, nanotube arrays, in situ synchronous anodization

## Abstract

**Highlights:**

**What are the main findings?**

- A one-step in situ anodization method successfully fabricates Gd-doped TiO_2_ nanotubes with uniform dopant distribution.
- Gd doping narrows TiO_2_’s bandgap to 2.46 eV, extending visible-light absorption beyond 500 nm.
- The Gd-TiO_2_ nanotubes degrade 90% methylene blue in 60 min, 50% more efficient than undoped TiO_2_.

**What are the implications of the main findings?**

- Simplifies the synthesis of rare-earth-doped TiO_2_, reducing costs.
- Enhances solar energy utilization of TiO_2_-based photocatalysts for broader environmental remediation applications.
- Provides insights into bandgap engineering via rare earth doping for designing high-performance photocatalysts.

**Abstract:**

Traditional methods for preparing rare-earth-doped TiO_2_ nanotubes are multi-step and often result in uneven dopant distribution, while pure TiO_2_ is limited by its wide bandgap and rapid charge recombination. In this study, a one-step in situ synchronous anodization strategy is developed to fabricate gadolinium (Gd)-doped TiO_2_ nanotube arrays directly on a titanium substrate. By adding gadolinium nitrate to an ethylene glycol–NH_4_F electrolyte, Gd incorporation and nanotube growth are achieved simultaneously, reducing the processing steps by over 60%. The obtained Gd–TiO_2_ nanotubes exhibit extended visible-light absorption with an edge beyond 500 nm and show a methylene blue degradation efficiency of 90% within 60 min, which is 50% higher than that of undoped TiO_2_. Scavenger experiments reveal that ·OH radicals play the predominant role in the photocatalytic process. First-principles calculations further confirm significant bandgap narrowing from 2.89 eV to 2.46 eV after Gd doping. This work provides a simple, efficient, and scalable synthesis route for high-performance TiO_2_-based photocatalysts with enhanced solar-driven activity.

## 1. Introduction

Industrial wastewater containing refractory organic pollutants poses serious threats to aquatic ecosystems and human health [1,2]. Photocatalytic degradation has emerged as a promising solution, capable of mineralizing organic contaminants into harmless small molecules under mild conditions [3,4]. Among various photocatalysts, TiO_2_ is widely studied due to its high chemical stability, low cost, and non-toxicity [5]. Since the initial report on its photocatalytic water-splitting activity [6], TiO_2_ has been extensively explored for environmental remediation [7]. However, its practical application is hindered by two inherent limitations: a wide band gap (~3.2 eV for anatase), which restricts light absorption mainly to the UV region (~5% of solar spectrum), and a rapid recombination rate of photogenerated electron–hole pairs, leading to low quantum efficiency [8,9].

To address these issues, doping with rare earth elements has been proposed as an effective modification strategy [10]. The introduction of rare earth ions can create defect levels within the TiO_2_ lattice, narrowing the band gap and suppressing charge carrier recombination [11]. In particular, gadolinium (Gd) has shown notable potential owing to its unique 4f electron configuration, which can enhance electron trapping and extend photo-response [1,12]. Conventionally, Gd-doped TiO_2_ nanotubes are fabricated via multi-step processes. For instance, several studies first prepare pure TiO_2_ nanotubes by anodic oxidation, followed by secondary treatments such as impregnation [13], electrochemical deposition [14], or sol–gel coating to introduce Gd species. Although these methods can successfully incorporate rare earth elements [15,16], they often result in non-uniform dopant distribution, require prolonged processing time, and involve complex procedures that increase production costs—factors that collectively limit their scalability for industrial applications [17]. Furthermore, the precise role of Gd in modulating the electronic structure and photocatalytic activity of TiO_2_ remains inadequately understood, with mechanistic insights still lacking [18,19].

Nanostructured TiO_2_ in the form of highly ordered nanotube arrays offers intrinsic advantages, including a large specific surface area for reactant adsorption and a directional architecture that facilitates charge transport [6,20]. To overcome the drawbacks associated with conventional multi-step doping routes, we propose a novel one-step synthesis strategy: in situ synchronous anodization using a Gd-containing electrolyte. This approach enables the simultaneous growth of TiO_2_ nanotubes and incorporation of Gd dopants during the anodization process [21]. Compared with existing methods, our technique ensures more homogeneous dopant distribution, shortens the fabrication cycle, reduces cost, and is readily scalable. Moreover, this work systematically investigates the underlying mechanism through which Gd doping enhances photocatalytic performance, providing new insights into the design of efficient rare earth-modified TiO_2_ nanomaterials [22].

## 2. Materials and Methods

### 2.1. Materials and Reagents

**Figure 1:**
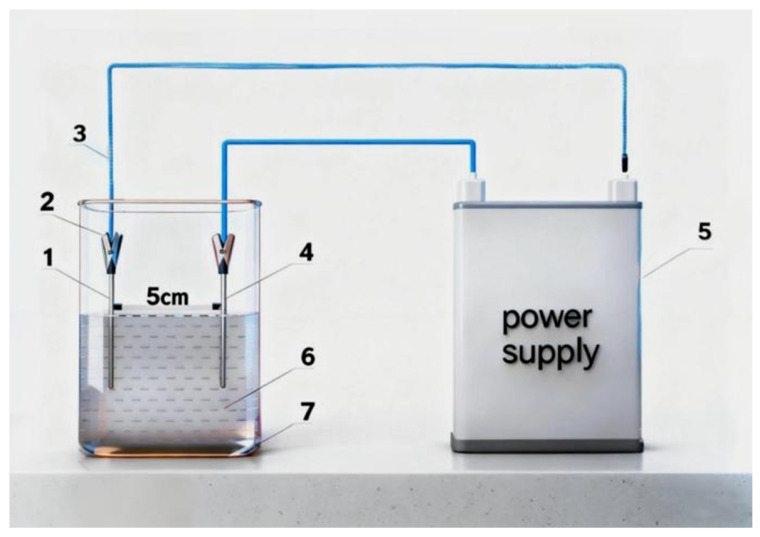
Schematic Diagram of the Anodization Setup: 1. cathode plate; 2. fixture; 3. wire; 4. anode; 5. power supply; 6. electrolyte; 7. beaker.

All chemicals utilized in this study were of analytical grade. Commercial pure titanium (CP-Ti) sheets served as the anode substrate. Gadolinium nitrate hexahydrate (Gd(NO_3_)_3_·6H_2_O), citric acid monohydrate (C_6_H_8_O_7_·H_2_O), ammonium fluoride (NH_4_F), ethylene glycol (EG), anhydrous ethanol, methylene blue (MB), terephthalic acid (TA), isopropanol (IPA), disodium ethylenediaminetetraacetate (EDTA-2Na), and L-histidine were used as received. Deionized water (DI water) was used in all experiments. Gd-doped TiO_2_ nanotube arrays (Gd-TNTAs) were fabricated via a one-step anodization approach. Prior to anodization, the CP-Ti sheets were cut into specified dimensions, mechanically polished, and subsequently cleaned by sequential immersion in alkaline and acidic solutions, followed by ultrasonication in ethanol and DI water. After drying under a nitrogen stream, the samples were stored in a vacuum desiccator to prevent surface oxidation. The anodization electrolyte was prepared as a low-fluorine, environmentally benign mixture. According to the stoichiometric design, 0.634 g of citric acid monohydrate, 3.99 g of gadolinium nitrate hexahydrate, and 0.09 g of ammonium fluoride were dissolved in a mixed solvent of DI water and ethylene glycol (volume ratio 1:4) under continuous magnetic stirring for 30 min to ensure complete homogenization. Anodization was carried out in a two-electrode configuration using a nickel sheet as the cathode and the pretreated CP-Ti sheet as the anode. A constant DC voltage of 30 V was applied for 2 h at room temperature using a DC power supply (PSW250-9, GW Instek, New Taipei city, Taiwan). The process schematic is illustrated in Figure 1. After anodization, the samples were thoroughly rinsed with DI water and ethanol to remove residual electrolyte. Finally, the as-prepared samples were annealed at 550 °C for 2 h under a continuous argon flow, followed by furnace cooling to room temperature to obtain crystallized Gd-doped TiO_2_ nanotube arrays. For comparative purposes, La-doped TiO_2_ (La-TiO_2_) and Ce-doped TiO_2_ (Ce-TiO_2_) nanotube arrays were prepared using the identical one-step anodization protocol, with Gd(NO_3_)_3_·6H_2_O replaced by equimolar La(NO_3_)_3_·6H_2_O and Ce(NO_3_)_3_·6H_2_O, respectively.

### 2.2. Characterization Methods

The crystalline phases of the samples were determined by X-ray diffraction (PANalytical Empyrean, Almelo, The Netherland). Morphology and elemental composition were examined using field-emission scanning electron microscopy (Hitachi High-Tech corporation, Hitachi city, Japan) coupled with energy-dispersive X-ray spectroscopy (EDS). Chemical states and surface composition were analyzed by X-ray photoelectron spectroscopy (ULVAC-PHI Inc., Chigasaki, Japan), with elemental concentrations calculated from peak areas using MultiPak software v. 9.9.0 (ULVAC-PHI, Inc., Chigasaki, Japan). Optical absorption properties were measured by UV-Vis spectrophotometry (Shimadzu Corporation, Kyoto, Japan).

### 2.3. Photocatalytic Testing

All photocatalytic tests were carried out in a closed reaction system (Beijing China Education light Source Technology Co., Ltd., Beijing, China) equipped with a cooling water circulator. The distance between the lamp and the reactor was set at 10 cm. When a 300 W xenon lamp was used without any filter (to simulate solar light), the corresponding light intensity at this distance was approximately 50–100 mW/cm^2^. After installing a near-infrared (NIR) long-pass filter (λ > 780 nm), the light intensity at the same distance decreased to 20–50 mW/cm^2^. This is because the photon energy of NIR light is relatively low, resulting in a weaker irradiation intensity compared with that of full-spectrum simulated solar light. The photocatalytic activity was evaluated by degrading methylene blue (MB, 10 mg L^−1^) under irradiation. In a typical run, the photocatalyst was immersed in 100 mL of MB solution and magnetically stirred in the dark for 30 min to establish adsorption–desorption equilibrium [8]. The solution was then exposed to light from a 300 W xenon lamp. For simulated solar light, no filter was used; for near-infrared (NIR) light, a long-pass optical filter (λ > 780 nm) was placed in front of the lamp. During irradiation, aliquots were withdrawn at given time intervals, and the concentration of MB was determined from its absorbance measured with a UV-Vis spectrophotometer (Shimadzu Corporation, Kyoto, Japan). To identify the dominant active species in the photocatalytic process, scavenger experiments were performed by adding benzoquinone (BQ), disodium ethylenediaminetetraacetate (EDTA-2Na), or isopropanol (IPA) to quench superoxide radicals (O_2_^−^), holes (h^+^), and hydroxyl radicals (·OH), respectively. All photocatalytic runs were conducted under identical conditions unless otherwise specified. Additionally, first-principles calculations were carried out to obtain the electronic band structure and bandgap of the materials [23].

## 3. Results and Discussion

### 3.1. Structural and Morphological Characteristics

Before refinement, the crystal systems and space groups of each TiO_2_ phase were defined as references. Anatase belongs to the tetragonal system (I4_1_/AMD, No.141), and rutile also has a tetragonal structure (P4_2_/mnm, No.136). Both are typical structural parameters of TiO_2_ [24,25]. For Gd-doped samples, crystal system models of Gd-based impurities (e.g., Gd_2_O_3_ with cubic system Ia-3) were not introduced throughout the refinement process. Subsequent attempts to incorporate Gd_2_O_3_ into the fitting showed its diffraction peak contribution was <1%. It also increased the Rwp (weighted residual variance) from 7.2% to 10.5%, exceeding the literature-recognized standard of <8% [26,27]. This confirms the absence of independent Gd-based impurities in the samples, laying a model foundation for “lattice doping” rather than “phase segregation”.

**Figure 2:**
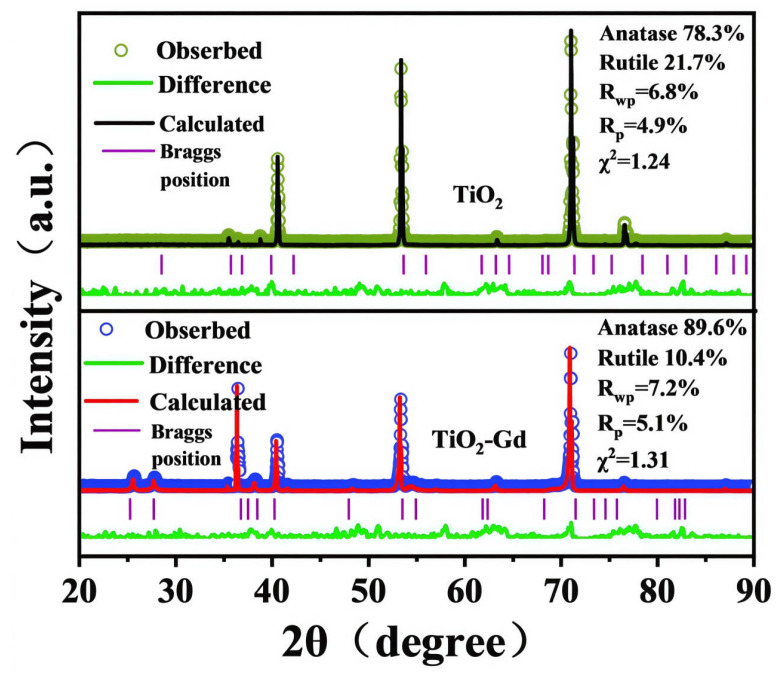
XRD patterns of TiO_2_ and TiO_2_-Gd.

Figure 2, showing quantitative Rietveld refinement analysis, revealed only two TiO_2_ phases (anatase and rutile) in the Gd-doped sample. No Gd-based impurities such as Gd_2_O_3_ or Gd(OH)_3_ were detected, with a fitted mass fraction of 0. Compared with the undoped sample, the anatase mass fraction increased from 78.3% to 89.6% after Gd doping. Meanwhile, the rutile proportion decreased from 21.7% to 10.4%. This indicates that the introduction of Gd^3+^ inhibits the anatase-to-rutile phase transformation. It further confirms that Gd is incorporated into TiO_2_ in the form of “lattice doping” rather than forming independent impurities to consume Gd sources.

Meanwhile, the lattice parameters, lattice volumes, and volume change rates of anatase and rutile TiO_2_ before and after Gd doping were obtained via XRD Rietveld refinement, respectively. For the anatase phase TiO_2_, the lattice parameters of the undoped sample were a = 3.785 ± 0.002 Å and c = 9.514 ± 0.003 Å, with a corresponding lattice volume of 339.2 ± 0.5 Å^3^. After Gd doping, the lattice parameter a increased to 3.798 ± 0.002 Å and c increased to 9.552 ± 0.003 Å. The lattice volume rose to 343.8 ± 0.5 Å^3^, with a volume change rate of +1.36%. For the rutile phase TiO_2_, the lattice parameters of the undoped sample were a = 4.594 ± 0.001 Å and c = 2.958 ± 0.001 Å, with a lattice volume of 62.4 ± 0.1 Å^3^. After Gd doping, the lattice parameter increased to 4.601 ± 0.001 Å and c increased to 2.965 ± 0.001 Å. The lattice volume increased to 62.8 ± 0.1 Å^3^, with a volume change rate of +0.64%.

The above results indicate that Gd doping can induce lattice expansion of TiO_2_, characterized by synchronous increases in lattice parameters a, c and lattice volume. This phenomenon can be attributed to the radius difference between doped and matrix ions. The ionic radius of Gd^3+^ (0.938 Å) is larger than that of Ti^4+^ (0.605 Å). When Gd^3+^ substitutes for Ti^4+^ sites in the lattice, it exerts a compressive effect on the surrounding lattice, leading to lattice distortion and volume expansion.

**Figure 3:**
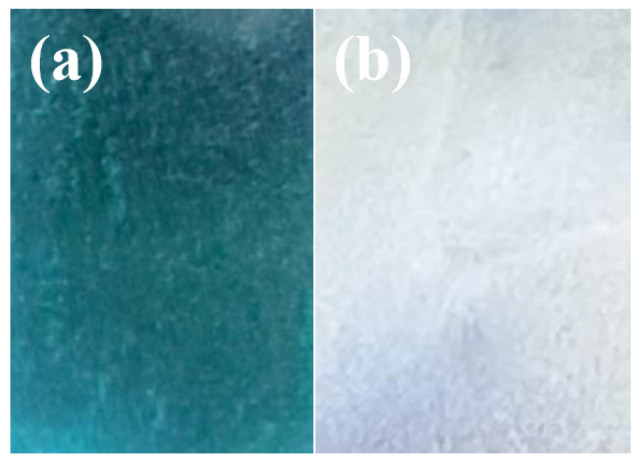
Macroscopic morphology of TiO_2_-Gd and TiO_2_. (**a**) TiO_2_-Gd. (**b**) TiO_2_.

Figure 3 presents the macroscopic surface morphology of the TiO_2_ and TiO_2_-Gd coatings. The TiO_2_-Gd sample exhibits a light blue, smooth surface (Figure 3a), in contrast to the silver-white appearance of the undoped TiO_2_ coating (Figure 3b). This color variation is attributed to the optical properties inherent to the materials. Pure TiO_2_ is a wide-bandgap semiconductor (≈3.2 eV for anatase), which primarily absorbs ultraviolet light (wavelength < ~387 nm) and thus appears transparent or silver-white in the visible region [1,6]. Although Gd^3+^ ions are colorless, their incorporation into the TiO_2_ lattice induces significant structural modifications [28]. The substitution of Ti^4+^ (ionic radius ≈ 0.605 Å) with larger Gd^3+^ ions (ionic radius ≈ 0.94 Å) creates lattice distortion [29]. To compensate for charge imbalance and alleviate structural strain, oxygen vacancies are generated [30]. These vacancies form defect energy levels within the bandgap, which may facilitate weak absorption in the blue-light region, resulting in the observed faint yellowish-blue tint in the Gd-doped sample [31].

**Figure 4:**
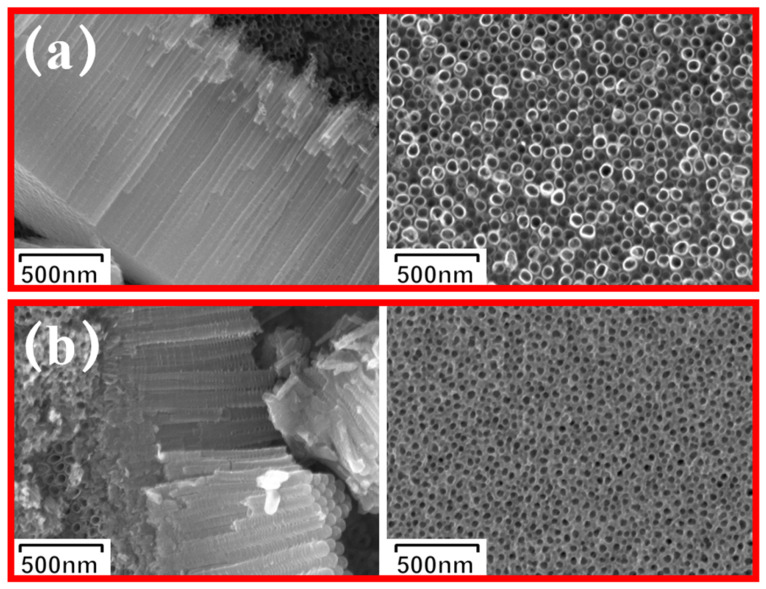

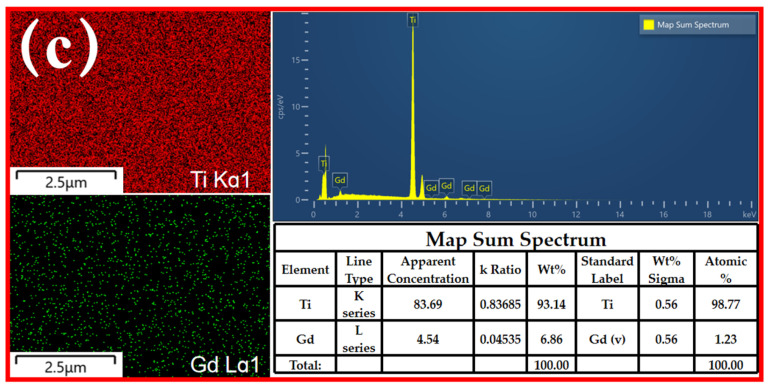
SEM images of TiO_2_-Gd and TiO_2_. (**a**) undoped nanotube arrays; (**b**) Gd-doped nanotube arrays; (**c**) EDS analysis of the TiO_2_-Gd sample.

Figure 4a and Figure 4b present the surface morphologies of undoped and Gd-doped TiO_2_ nanotube arrays, respectively. A clear comparison shows that the TiO_2_-Gd arrays possess a rougher surface texture than their undoped counterpart. This increased roughness is expected to provide a larger specific surface area, thereby contributing to enhanced photocatalytic activity, This is basically consistent with the viewpoint mentioned in the literature [32]. Figure 4c presents EDS elemental mapping and spectrum analysis of the TiO_2_-Gd sample. The upper (red) map shows Ti (Ti Kα1) distribution, and the lower (green) map shows Gd (Gd Lα1) distribution, both with a 2.5 μm scale bar. Overlapping, agglomeration-free distributions confirm uniform Gd dispersion in the TiO_2_ matrix at the micrometer scale. The right-side total spectrum displays only characteristic X-ray peaks of Ti and Gd, with no impurity interference, verifying the sample’s primary composition. Elemental content data (table) shows Ti at 93.14 wt% (98.77 at.%) as the matrix and Gd at 6.86 wt% (1.23 at.%) as the dopant, confirming low-concentration, uniform Gd doping. Collectively, these results confirm Gd is uniformly incorporated into the TiO_2_ matrix without forming independent Gd-based impurities.

### 3.2. XPS Analysis

**Figure 5:**
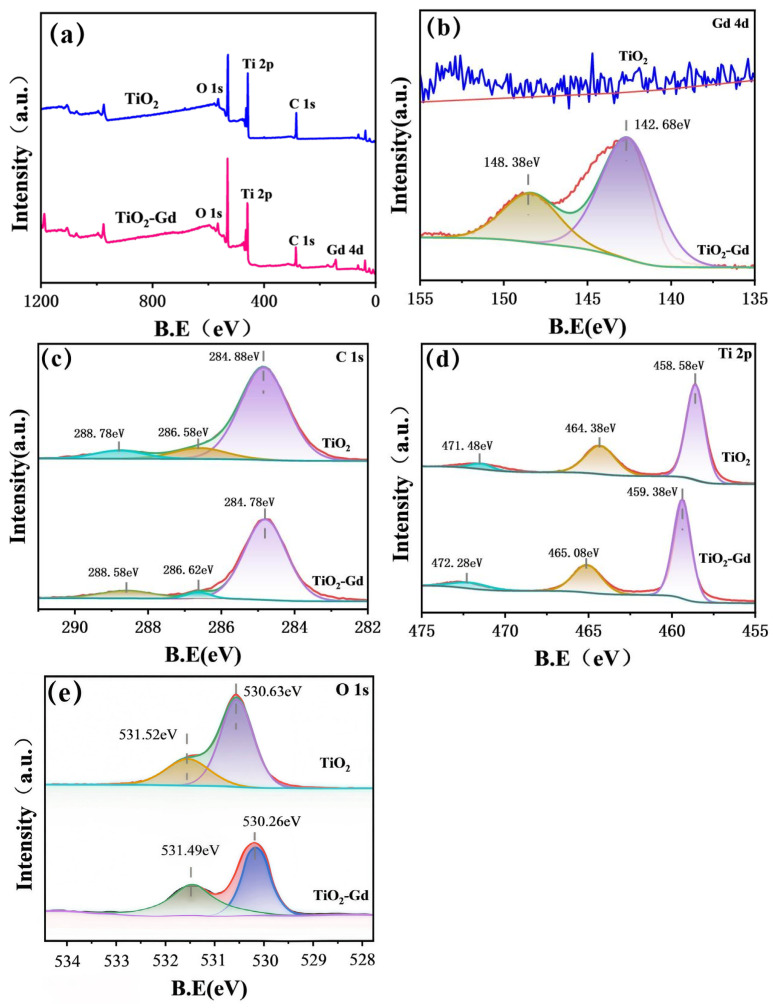
XPS spectra of TiO_2_-Gd and TiO_2_: (**a**) survey scan, (**b**) Gd 4d, (**c**) C 1s, (**d**) Ti 2p, (**e**) O 1s.

XPS analysis was performed to examine the oxidation states and chemical coordination in Gd-doped TiO_2_. Figure 5a displays characteristic peaks of Ti 2p, O 1s, C 1s, and notably Gd 4d, confirming the successful incorporation of Gd into the TiO_2_ structure [33]. Compared with pure TiO_2_, the Ti 2p region in the doped sample exhibits altered peak intensity along with a slight binding-energy shift, indicating that Gd doping modifies the local chemical environment of Ti [34]. The presence of Gd-related signals and the shift in Ti 2p collectively demonstrate effective doping and suggest changes in the electronic structure of TiO_2_ induced by Gd [11,35]. Thus, the XPS survey provides direct evidence of Gd doping and establishes a basis for understanding its influence on the photocatalytic properties of TiO_2_.

The Gd 4d XPS spectrum of the TiO_2_-Gd composite is presented in Figure 5b. Deconvolution of the spectrum reveals two primary contributions, centered at binding energies of approximately 148.38 eV and 142.68 eV, which are attributed to distinct electronic states of Gd [36]. These features indicate that Gd adopts two different chemical environments or oxidation states within the TiO_2_ matrix, likely arising from varied doping configurations such as differences in coordination geometry or local charge distribution [31]. The presence of multiple Gd states is significant, as it can directly modulate the physical and chemical properties of the material, including its optical response and surface catalytic behavior [37]. Furthermore, the incorporation of Gd introduces new electronic states associated with the Gd 4d orbitals, thereby modifying the host TiO_2_ electronic structure [11]. These induced states, located near the band edges, are expected to influence key processes such as photon absorption and the separation of photogenerated charge carriers [38]. Collectively, the XPS analysis confirms the successful doping of Gd into TiO_2_ and elucidates the specific chemical nature of the incorporated Gd species, providing insight into the structure–property relationships in this modified photocatalyst.

Figure 5c displays the Ti 2p XPS spectrum of pristine TiO_2_. Following spectral deconvolution, the spectrum is resolved into three constituent peaks. Consistent with spin–orbit coupling, the Ti 2p orbital manifests as the characteristic doublet, comprising the Ti 2p_3_/_2_ and Ti 2p_1_/_2_ components, observed at binding energies of approximately 458.58 eV and 464.38 eV, respectively [39]. An additional feature at approximately 471.48 eV is identified, which is typically ascribed to a satellite structure associated with the primary Ti 2p transitions [36]. The peak positions and spectral line shape are characteristic of Ti^4+^ in the TiO_2_ lattice, aligning well with established reference data [25]. In contrast, the Ti 2p spectrum of the Gd-doped sample (TiO_2_-Gd) exhibits a systematic positive shift upon deconvolution, with corresponding peaks located at approximately 459.38 eV (Ti 2p_3_/_2_), 465.08 eV (Ti 2p_1_/_2_), and 472.28 eV (satellite). This uniform shift to higher binding energy signifies an altered chemical environment for Ti following Gd incorporation [36]. The observed increase in Ti 2p binding energy is indicative of a reduced electron density around the Ti nuclei. This phenomenon can be attributed to the introduction of Gd, which, owing to its distinct electronic structure, engages in electronic interactions with the TiO_2_ host, effectively withdrawing electron density from neighboring Ti atoms [11]. Such a modification in the local electronic structure is a direct consequence of Gd integration into the TiO_2_ lattice and its interaction with the Ti–O framework [39]. The measurable shift in the Ti 2p core level thus provides direct evidence for the successful doping of Gd and its pivotal role in modulating the electronic properties of TiO_2_, which is intrinsically linked to the material’s functional performance, such as its catalytic activity [40].

Figure 5e shows the regulatory effect of Gd doping on oxygen defects. Gd substitutes for Ti sites in the lattice in the form of Gd^3+^ (ionic radius of Gd^3+^, 0.94 Å > 0.605 Å of Ti^4+^). To balance the charge imbalance, oxygen vacancies (oxygen defects) need to be generated in the lattice for compensation. Through the comparison of peak areas, the ratio of oxygen defects to adsorbed oxygen increased from 29% to 38%. This change also laid a structural foundation for the performance optimization of the samples.

### 3.3. Optical Properties

**Figure 6:**
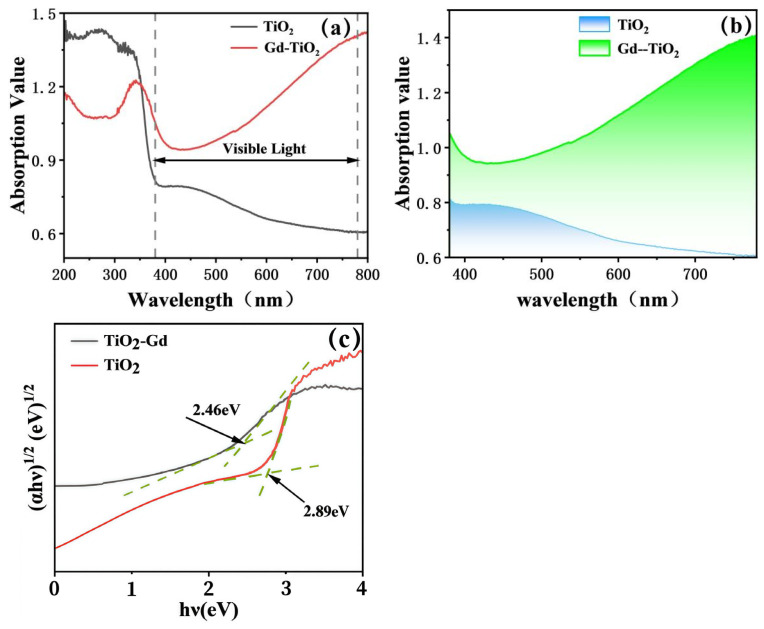
UV–vis diffuse reflectance spectra of (**a**) TiO_2_ doped with various rare-earth elements, (**b**) pure and Gd-doped TiO_2_ in the visible region, (**c**) Tauc plot of UV-Vis DRS for TiO_2_ and TiO_2_-Gd.

The optical absorption properties of the synthesized composites were investigated by UV-Vis absorbance spectra, as presented in Figure 6a,b. Pristine TiO_2_ displays a fundamental absorption edge near 400 nm, exhibiting strong absorption in the UV region but negligible absorption at wavelengths longer than 400 nm. This characteristic is attributed to the intrinsic wide bandgap of TiO_2_ (≈3.2 eV), which permits only the excitation of electrons by high-energy UV photons [1,6]. In contrast, a distinct redshift in the absorption edge and enhanced absorption in the visible-light range are observed for all rare-earth-doped samples. La-doped TiO_2_ demonstrates moderate redshift and improved visible-light absorption, indicating a reduction in the effective bandgap due to La incorporation, which extends the photoresponse range [41]. A more pronounced effect is seen for Ce-doped TiO_2_, which exhibits a further redshift and stronger visible-light absorption, suggesting that Ce doping mediates a more substantial bandgap modification. Notably, Gd-doped TiO_2_ presents the most significant optical alteration among the doped series. It possesses the broadest and most intense absorption profile across the entire visible spectrum (400–800 nm), implying that Gd doping induces the most effective bandgap narrowing and electronic structure modulation, thereby granting the composite superior visible-light harvesting capability. This enhanced visible-light absorption is of paramount importance for photocatalysis, as visible light constitutes a major portion of the solar spectrum [42]. The extended photoresponse in these doped TiO_2_ composites enables more efficient utilization of solar energy, which is anticipated to yield superior photocatalytic performance [43]. The increased photon absorption promotes the generation of a greater number of photogenerated electron-hole pairs, consequently supplying more charge carriers to drive surface redox reactions [44]. The optical band gaps of the samples were estimated via the Tauc relation based on the data from UV-Vis diffuse reflectance spectroscopy (DRS, diffuse reflectance mode) (Figure 6c). As TiO_2_ is an indirect band gap semiconductor, its Tauc relation requires modification in combination with the characteristics of diffuse reflectance data. The samples in this study were TiO_2_ nanotube films grown on opaque titanium substrates, making the absorption coefficient calculation method in transmission mode inapplicable. Thus, the diffuse reflectance (R) was converted to an equivalent absorption parameter F(R) (i.e., Kubelka-Munk function value) using the Kubelka-Munk function, which can replace the absorption coefficient α for band gap analysis. The expression of the Kubelka-Munk function is as follows: F(R) = (1 − R)^2^/2R, where R is the diffuse reflectance of the sample (directly measured by UV-Vis DRS), and F(R) reflects the combined effects of light absorption and scattering by the material.

The Tauc relation for indirect band gap semiconductors is modified to: (F(R)⋅hν)^1/2^∝(hν − Eg), where hν is the photon energy (converted from the wavelength λ via hν = 1240/λ, unit: eV) and Eg is the optical band gap. A plot of (F(R)⋅hν)^1/2^ versus hν was constructed (i.e., the Tauc plot in Figure 6c). Tangents were drawn to the linear segments of the curve, and the hν value corresponding to the intersection of the two tangents was defined as the optical band gap of the sample: the band gap of pure TiO_2_ was 2.89 eV, while that of Gd-doped TiO_2_ (Gd-TiO_2_) was narrowed to 2.46 eV.

This band gap narrowing is consistent with the enhanced visible-light absorption of Gd-TiO_2_ in Figure 6a. The reason is that Gd doping introduces impurity energy levels into the TiO_2_ lattice, reducing the energy threshold for electron transitions. This structural change not only narrows the band gap but also boosts the material’s visible-light absorption capacity, laying a structural foundation for the visible-light-driven photocatalytic performance of Gd-TiO_2_.

### 3.4. Photocatalytic Performance

An orthogonal experimental design was employed to identify appropriate electrolyte parameters. In this study, the concentration gradient of Gd(NO_3_)_3_·6H_2_O was set as 0 (not added), 0.01 mol/L, and 0.03 mol/L. This gradient design is based on the following scientific bases.

It serves as an experimental benchmark. It clearly distinguishes the differences in lattice structure, oxygen defect evolution, and final performance between doped and undoped samples. It is a core reference for verifying the regulatory effect of Gd doping. It can directly quantify the impact of doping behavior on the material. From the perspective of doping mechanism, the ionic radius of Gd^3+^ (0.94 Å) is significantly larger than that of Ti^4+^ (0.605 Å). Low-concentration doping can introduce controllable lattice distortion and oxygen vacancies. Meanwhile, it avoids the risk of lattice collapse or phase separation caused by high-concentration doping. From the perspective of academic relevance, 0.01 mol/L is a classic low-concentration range in TiO_2_ rare earth doping research. It is highly consistent with the concentration range (0.001–0.05 mol/L) reported in existing studies [16]. This facilitates the horizontal comparison of experimental results and academic exchanges. From the analysis of dosage effect, this concentration forms a continuous gradient with 0.01 mol/L. It allows systematic study of the correlation between doping concentration, lattice expansion, and oxygen defect ratio. It helps identify the doping concentration range with optimal performance. From the perspective of stability balance, the medium concentration can ensure sufficient Gd^3+^ to replace Ti^4+^ lattice sites. This effectively introduces oxygen vacancies. It also avoids the increase in carrier recombination centers caused by excessive defects. It is a key concentration for balancing defect regulation and lattice stability. From the perspective of synthesis feasibility, in the anodic oxidation synthesis system, the concentration of 0.03 mol/L can ensure the uniform dispersion of the precursor solution. It avoids local agglomeration or precipitation caused by excessively high concentration [34]. This guarantees the uniformity of the samples.

**Figure 7:**
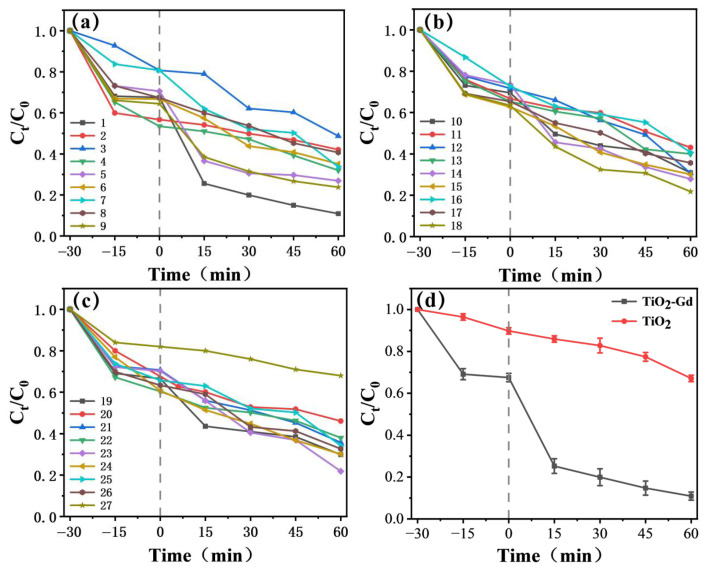
(**a**–**c**) Photocatalytic degradation rates of MB for Groups 1-27 of TiO_2_-Gd; (**d**) Comparison of degradation rates between TiO_2_-Gd and TiO_2_.

This concentration gradient covers the typical range of “undoped—low doping- medium doping”. It can clearly reveal the structure-activity relationship between Gd doping concentration, material structure, defects, and performance. It provides a reliable experimental basis for the subsequent optimization of doping strategies. As detailed in Table 1 and Table 2, the photocatalytic activity was assessed by monitoring the photodegradation efficiency of methylene blue (MB) under simulated sunlight irradiation using a CEL-HXF300 photocatalytic reaction system. Compared to pristine TiO_2_, the Gd-doped sample (TiO_2_-Gd) exhibited markedly enhanced degradation performance. As depicted in Figure 7d, the normalized concentration (Cₜ/C_0_) of MB decreased rapidly with irradiation time. A particularly sharp decline was observed within the initial 15 min, followed by a continued rapid reduction. After 60 min of reaction, Cₜ/C_0_ reached approximately 0.1, indicating significantly faster and more effective pollutant degradation by TiO_2_-Gd [44].

Consistent with the UV-Vis analysis, Gd doping extends the light absorption of TiO_2_ into the visible region. This enhanced photon harvesting capability enables TiO_2_-Gd to utilize a broader spectrum of solar energy, thereby generating a greater number of photogenerated electron–hole pairs [45]. These charge carriers serve as active species that drive subsequent degradation reactions [46]. Furthermore, the incorporation of Gd modifies the electronic structure of TiO_2_, which facilitates the separation of photogenerated charges and suppresses their recombination. The increased availability of these electrons and holes significantly improves the overall efficiency of the redox processes involved in pollutant degradation [47].

Surface morphology plays a critical role in catalytic performance. While smooth surfaces offer limited active area, rough surfaces characterized by nanoscale pores, steps, and edges provide a substantially increased specific surface area. This is consistent with the SEM results presented in the previous section. This enlarged surface offers more active sites for the adsorption of reactant molecules (e.g., pollutants and water), thereby establishing more venues for photocatalytic reactions and forming the physical basis for enhanced efficiency [48].

Moreover, rough surfaces promote the “light-trapping effect.” In contrast to the specular reflection prevalent on smooth surfaces, which leads to considerable photon loss, rough surfaces induce multiple internal reflections and scattering of incident light [49]. This phenomenon prolongs the optical path length within the material, markedly boosting photon absorption and utilization. Consequently, more electron–hole pairs are generated, directly amplifying the driving force for photocatalysis [50]. (Table 1 and Table 2)

**Figure 8:**
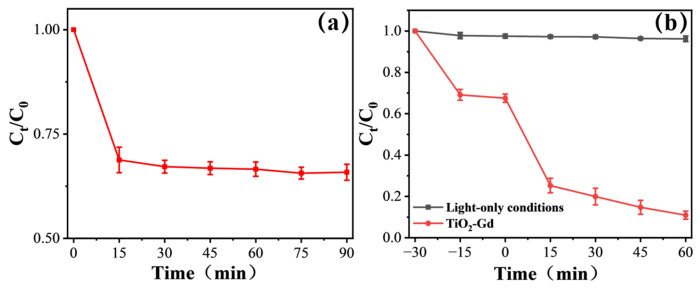
(**a**) TiO_2_-Gd adsorption–desorption curve. (**b**) Comparison of MB degradation rates under light-only condition and with TiO_2_-Gd addition.

Figure 8a shows the variation in MB concentration over time in a dark environment. In the initial stage (0–20 min), the MB concentration decreases slightly due to adsorption on the catalyst surface. After 25 min, the curve levels off, with the C/C_0_ value stabilizing at around 0.95 without significant fluctuations. This trend indicates that 30 min of dark stirring is sufficient for MB molecules to establish a dynamic adsorption–desorption equilibrium between the TiO_2_-Gd catalyst surface and the solution. The impact of the adsorption process on MB concentration reaches a steady state. At equilibrium, the adsorption removal rate of MB by TiO_2_-Gd is only about 5%, much lower than the 90% degradation rate after 60 min of photocatalytic reaction. This suggests that the significant decrease in MB concentration during the subsequent light irradiation stage is mainly attributed to photocatalytic degradation rather than the continuous contribution of adsorption. It eliminates the interference of unbalanced adsorption on the evaluation of photocatalytic activity.

As shown in Figure 8b, throughout the 60 min light irradiation cycle, the C/C_0_ value of the “light-only condition” curve remains above 0.98. No obvious decrease in MB concentration is observed, with a degradation rate of less than 2%. This result fully confirms that MB molecules exhibit excellent photostability under the simulated sunlight irradiation used in the experiment and do not undergo photodegradation on their own.

**Figure 9:**
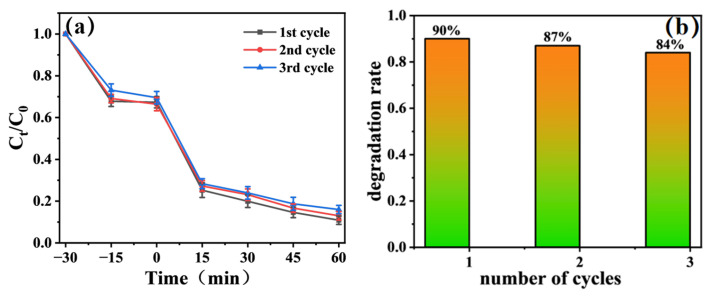
(**a**) Time-dependent normalized concentration (Ct/C0) curves of MB degradation by TiO_2_-Gd over three consecutive cycles; (**b**) Degradation Efficiency of TiO_2_-Gd in Three-cycle Experiments.

Figure 9a depicts the time-normalized concentration (C_t_/C_0_) profiles of methylene blue (MB) degradation over three consecutive cycles using Gd-doped TiO_2_ (TiO_2_-Gd) as the photocatalyst.

The degradation curves exhibit highly consistent trends across all three cycles: the MB concentration declines sharply within the initial 15 min of light irradiation, followed by a relatively slow and steady degradation stage. After 60 min of illumination, the C_t_/C_0_ value stabilizes at approximately 0.1 for each cycle, corresponding to a degradation efficiency of ~90%. Notably, no obvious upward shift in the curves is observed in subsequent cycles, indicating that the photocatalytic activity of TiO_2_-Gd does not undergo significant decay after washing treatments, and its degradation kinetic behavior remains stable. This favorable stability can be ascribed to the homogeneous dispersion of Gd^3+^ ions within the TiO_2_ lattice via the in situ anodization process. Specifically, no discrete Gd-based crystalline phases are formed; instead, Gd ions establish strong chemical bonds with the Ti–O framework, which effectively inhibits the leaching or agglomeration of active components during cyclic testing [36].

Figure 9b presents the histogram of MB degradation efficiency for TiO_2_-Gd over three consecutive cycles, which intuitively quantifies the extent of performance attenuation during cyclic operation. Statistical results reveal that the degradation efficiency of TiO_2_-Gd only decreases slightly from the initial 90% to 84% after three cycles, corresponding to a total attenuation of merely 6%. This result confirms the excellent cyclic stability of TiO_2_-Gd. The underlying mechanism is associated with the defect energy levels and oxygen vacancies introduced by Gd doping: these structural modifications can continuously modulate the electronic structure of TiO_2_, promote the separation of photogenerated electron–hole pairs, and ensure the sustained generation of hydroxyl radicals (·OH), the dominant active species responsible for MB degradation. Consequently, the attenuation of catalytic efficiency induced by the reduced yield of active species is effectively mitigated [41].

**Figure 10:**
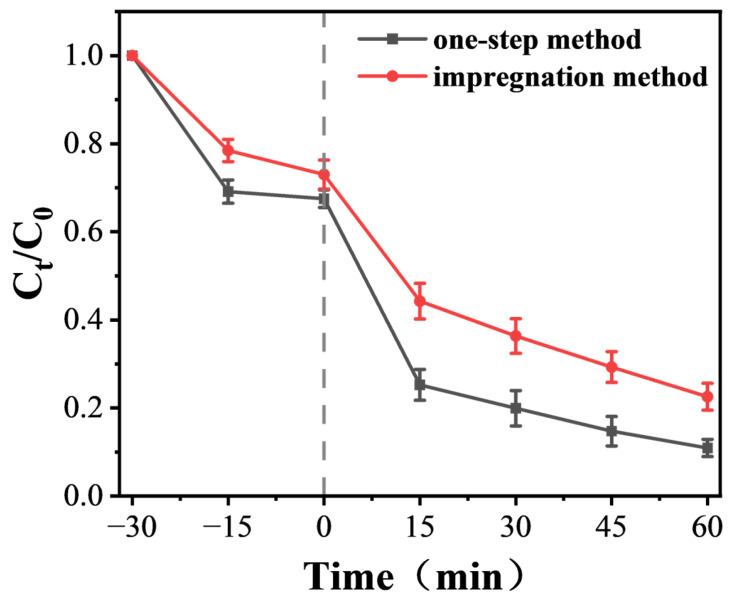
Comparison of Degradation Performance of Samples Synthesized by One-Step Method and Traditional Impregnation Method.

Figure 10 compares the degradation performance of samples doped by the one-step method and the traditional impregnation method by tracking the variation trend of C_t_/C_0_.

As can be seen from the curve trends in Figure 10, in the initial stage of the reaction, the C_t_/C_0_ value of the sample prepared by the one-step method decreases significantly faster than that of the sample prepared by the impregnation method. This indicates that the sample doped via the one-step method can exert its effect rapidly at the initial degradation stage and exhibits superior initial degradation efficiency.

Throughout the entire reaction cycle, the C_t_/C_0_ value of the one-step method sample remains consistently lower than that of the impregnation method sample, and the gap between the two gradually widens with the passage of time. This result directly demonstrates that the degradation performance of the sample prepared by the one-step method is superior to that of the sample prepared by the traditional impregnation method during the whole reaction process, which also highlights the advantage of the one-step method in enhancing the degradation activity of the samples.

### 3.5. Simulation Calculations

Titanium dioxide (TiO_2_), a widely studied semiconductor, exists primarily in three crystalline phases: brookite, rutile, and anatase. These phases exhibit distinct unit cell structures and bandgap energies, which fundamentally govern their physical and chemical properties [36,39]. To elucidate the mechanism by which Gd doping enhances the photocatalytic performance of TiO_2_, computational simulations of these three phases were performed using Materials Studio [23]. The calculations were based on the Perdew-Burke-Ernzerhof (PBE) exchange-correlation functional within the GGA framework. To address the intrinsic underestimation of bandgaps by pure GGA and properly describe the localized d/f orbitals, Hubbard U corrections were applied: U(Ti-3d) = 4.2 eV (a widely adopted value for anatase TiO_2_ to match experimental structural and electronic properties [36,39]) and U(Gd-4f) = 6.0 eV (selected to account for the strong correlation of Gd-4f electrons, consistent with previous rare-earth doping studies [28,35]). The electron-ion interactions were described using ultrasoft pseudopotentials, with a cutoff energy of 400 eV set for the plane-wave basis set to ensure numerical convergence. For geometric optimization, the conjugate gradient algorithm was used, with convergence criteria set to 1 × 10^−5^ eV per atom for total energy and 0.02 eV/Å for atomic forces. For electronic structure calculations, a Monkhorst-Pack k-point grid of 3 × 3 × 1 was sampled for the anatase TiO_2_ unit cell (and corresponding supercell for Gd doping) to ensure sufficient sampling of the Brillouin zone [51].

**Figure 11:**
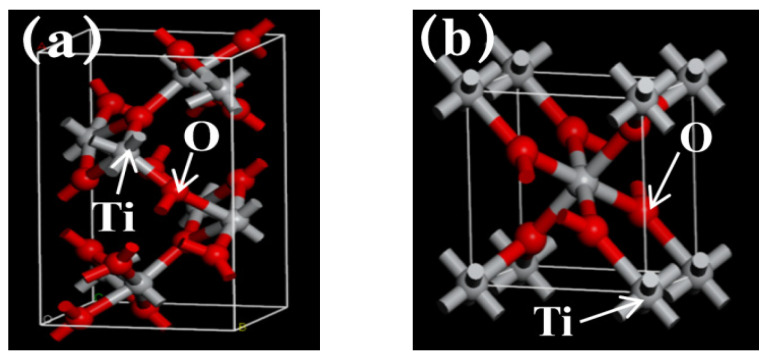

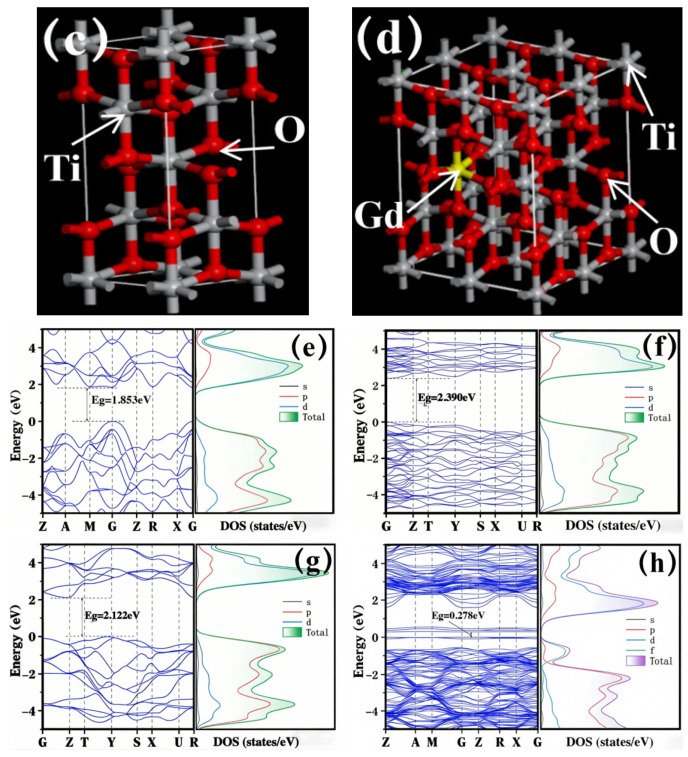
Computational models and calculated bandgaps. (**a**–**c**) Unit cells of brookite, rutile, and anatase TiO_2_. (**d**) Supercell of Gd-doped anatase. (**e**–**g**) Corresponding bandgap energies of pure TiO_2_ phases obtained from GGA+U calculations (U(Ti-3d) = 4.2 eV). (**h**) Bandgap energy of Gd-doped anatase TiO_2_ obtained from GGA+U calculations (U(Ti-3d) = 4.2 eV, U(Gd-4f) = 6.0 eV).

Figure 11a–d show the unit cells of four crystals, respectively. As illustrated in Figure 11a–d, brookite crystallizes in the orthorhombic system, while both rutile and anatase adopt tetragonal structures [36]. The calculated bandgap energies, derived from MS simulations and presented in Figure 11e–g, are 2.390 eV for brookite, 1.853 eV for rutile, and 2.122 eV for anatase [36]. Notably, following the construction of a supercell and the introduction of Gd, the bandgap of anatase TiO_2_ decreased substantially to 0.278 eV (Figure 11h). This significant reduction is attributed to the introduction of new energy states within the intrinsic bandgap of TiO_2_ upon Gd doping, effectively narrowing the bandgap and inducing a redshift in the optical absorption edge [11,39]. Consequently, the material gains enhanced capacity to harvest visible light.

As reported by Reference [11], the incorporation of Gd modifies the electron density distribution surrounding atoms within the TiO_2_ lattice, thereby reducing the energy required for electron excitation from the valence band to the conduction band. Notably, this effect not only enhances the yield of photogenerated charge carriers but also accelerates their generation kinetics, which collectively contributes to improved photocatalytic efficiency. Furthermore, the impurity energy levels associated with Gd can act as trapping sites for either electrons or holes, facilitating the spatial separation of photogenerated electron–hole pairs while suppressing their recombination. Consequently, electrons localized at Gd-related states exhibit a substantially reduced probability of recombination with holes, enabling a greater number of charge carriers to migrate to the surface and participate in redox reactions [38]. Structurally, Gd doping may also modify the local crystal arrangement and surface atomic configuration of TiO_2_, creating additional defects such as oxygen vacancies and increasing the density of active sites. These sites often exhibit stronger affinity for pollutant molecules, improving adsorption on the catalyst surface [48]. The enhanced adsorption increases the contact probability between target pollutants and photogenerated carriers, thereby accelerating degradation rates [9].

As indicated by the EDS results presented above, the doping concentration of Gd atoms was approximately 1.23 at.%. We first optimized the three unit cells of pristine TiO_2_ (brookite, rutile, anatase) to obtain their ground-state structures. Subsequently, a 2 × 2 × 1 anatase supercell (containing 32 Ti atoms and 64 O atoms) was constructed for Gd doping. One Ti atom in the supercell was substituted with a Gd atom, corresponding to a doping concentration of approximately 1.04 at. The minor error ensures good agreement with the practical doping condition. The supercell was then re-optimized to relax the lattice distortion induced by the substitution of Ti^4+^ (ionic radius: 0.605 Å) with the larger-sized Gd^3+^ (ionic radius: 0.94 Å).

Despite these systematic deviations, the pronounced reduction in the calculated bandgap following Gd doping consistently indicates that the enhanced photocatalytic activity of TiO_2_-Gd is closely correlated with effective bandgap narrowing [19].

Notably, a clear discrepancy exists between first-principles calculated and experimentally measured band gaps, a common observation in semiconductor research. The key origins of this deviation are as follows.

First, DFT calculations with the GGA functional systematically underestimate semiconductor band gaps due to its approximate treatment of electron exchange-correlation energy. This leads to the calculated band gaps of undoped TiO_2_ (2.122 eV) and Gd-doped TiO_2_ (0.278 eV) being significantly lower than the experimental values of 2.89 eV and 2.46 eV, respectively. Furthermore, theoretical calculations rely on an idealized periodic supercell model assuming uniform dopant distribution and a defect-free lattice, whereas real samples contain oxygen vacancies, surface states, and grain boundaries. Local lattice distortion or Gd segregation induced by doping is also omitted in the model, contributing to electronic structure deviations. Finally, experimental band gaps derived from Tauc plot extrapolation are subject to errors from sample thickness, surface roughness, and diffuse scattering. Additionally, complex defect states (e.g., oxygen vacancies, hydroxyl groups) that introduce impurity levels in the band gap are simplified or neglected in calculations, further widening the theory-experiment gap.

Overall, this discrepancy arises from the inherent limitations of the GGA functional and the contrast between the idealized computational model and the structural complexity of real materials.

### 3.6. Reaction Mechanism

**Figure 12:**
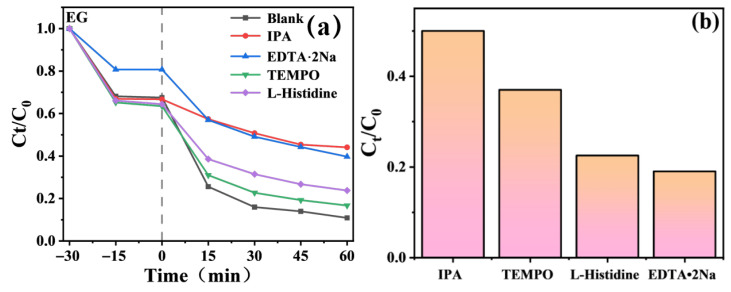
Scavenging experiment for active species during the photocatalytic degradation of MB. (**a**) Active substance concentration *vs* time; (**b**) Active substance contribution in MB photocatalytic degradation.

To further investigate the contribution of active species in the degradation process, active free radical trapping experiments were carried out. In the reaction system for the degradation of methylene blue (MB) solution by TiO_2_-Gd under simulated sunlight, disodium ethylenediaminetetraacetate (EDTA-2Na), L-histidine, 2,2,6,6-tetramethylpiperidin-1-oxyl (TEMPO) and isopropanol (IPA) were added separately, which selectively quench photogenerated holes (h^+^), singlet oxygen, superoxide radicals (·O_2_^−^) and hydroxyl radicals (·OH), respectively. As shown in Figure 12, the degradation rate of MB solution by TiO_2_-Gd only decreased slightly after the addition of EDTA-2Na, L-histidine and TEMPO, indicating that h^+^ and ·O_2_^−^ are not the primary active species in this photocatalytic process. In contrast, the photocatalytic degradation capacity was significantly reduced with the addition of IPA, which demonstrates that ·OH plays a pivotal role in the photocatalytic degradation process. This result is consistent with the high MB degradation efficiency observed in the oxygen-annealed TiO_2_ nanotube system.

This aligns with the high MB degradation efficiency observed in oxygen-annealed TiO_2_ nanotube systems. Specific reaction conditions (e.g., bias potential, electrolyte) were optimized to enhance active species generation, which is basically consistent with the viewpoint mentioned in the literature [13].

**Figure 13:**
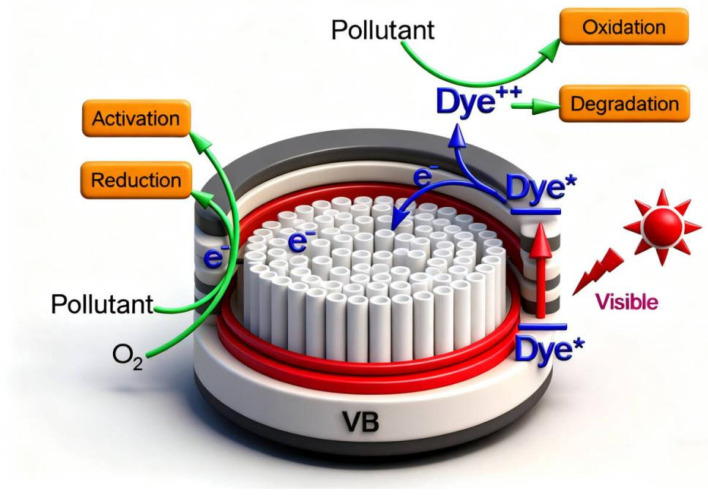
Mechanism for the TiO_2_-Gd photocatalyst.

Based on this, a possible mechanism for the TiO_2_-Gd photocatalyst was proposed, as illustrated in Figure 13. The process of TiO_2_ photocatalytic degradation of pollutants begins with its light absorption characteristics as a semiconductor. When incident photons have energy equal to or greater than the bandgap of TiO_2_ (≈3.2 eV, corresponding to ultraviolet light) [1,6], electrons (e^−^) in the valence band (VB) are excited to the conduction band (CB). Positively charged holes (h^+^) are left behind in the valence band. This generates highly active electron–hole pairs (e^−^-h^+^ pair). This initiates the entire reaction, described by the equation [47]:

TiO_2_ + hν → e_cb^−^ + h_vb^+^

The separation and migration of photogenerated electrons and holes are crucial for subsequent reactions. Separated carriers migrate to the surface of TiO_2_ particles. During this process, most recombine and deactivate [49]. Only a few successfully reach the surface to participate in reactions. So inhibiting recombination is key to improving photocatalytic efficiency [46]. Holes (h^+^) reaching the surface possess strong oxidizing ability. They can directly oxidize organic pollutants (R) adsorbed on the catalyst surface, degrading them: h^+^ + R → R^+^ → … → degradation products. However, the primary pathway involves holes reacting with water molecules (H_2_O) or hydroxyl groups (OH^−^) adsorbed on the catalyst surface. This generates hydroxyl radicals (·OH):

h_vb^+^ + H_2_O → ·OH + H^+^

h_vb^+^ + OH^−^ → ·OH

Simultaneously, electrons (e^−^) in the conduction band have strong reducing ability. They combine with oxygen (O_2_) molecules adsorbed on the TiO_2_ surface [2,50]. This reduces them to superoxide radical anions (·O_2_^−^):

e_cb^−^ + O_2_ → ·O_2_^−^

The generated ·O_2_^−^ can be protonated under acidic conditions. It forms hydroperoxyl radicals (·HO_2_), and ultimately converts into hydrogen peroxide (H_2_O_2_) and more hydroxyl radicals (·OH):

·O_2_^−^ + H^+^ → ·HO_2_

2·HO_2_ → O_2_ + H_2_O_2_

H_2_O_2_ + e_cb^−^ → ·OH + OH^−^

Thus, pollutant degradation is primarily accomplished through highly reactive free radicals (·OH) and a small amount of direct hole oxidation. Hydroxyl radicals can non-selectively attack chemical bonds in organic compounds. Through a series of complex chain reactions (e.g., decarboxylation, ring opening, bond breaking), they ultimately mineralize organics into carbon dioxide (CO_2_), water (H_2_O), and small inorganic ions [8,45]. The entire process of photocatalytic degradation is a complex redox reaction chain driven by light on the semiconductor surface. Its efficiency highly depends on the generation [2,8], separation, migration of photogenerated carriers, and the competition and synergy of surface reactions.

In summary, under light irradiation, TiO_2_-Gd absorbs photons. Valence band electrons are excited to the conduction band, generating photogenerated electrons and holes. Due to Gd doping, the recombination of photogenerated electron–hole pairs is effectively suppressed [11,38]. More photogenerated holes can migrate to the surface of TiO_2_-Gd. These photogenerated holes have strong oxidizing ability. On one hand, they can directly oxidize methylene blue (MB) molecules adsorbed on the catalyst surface [1,6]. On the other hand, they react with hydroxyl groups or water molecules on the TiO_2_-Gd surface, generating a large number of hydroxyl radicals [46]. The significant decline in degradation capability after adding isopropanol confirms that ·OH is the key active species in photocatalytic degradation of MB. It can decompose MB molecules into harmless small molecules through strong oxidation. Although photogenerated electrons also react with oxygen to form superoxide anion radicals, the degradation rate only slightly decreased after adding BQ. This indicates that superoxide radicals are not the primary active species. While photogenerated holes have direct oxidizing effects, the degradation rate only slightly decreased after adding EDTA-2Na and L-histidine. This suggests that direct electron–hole interactions are relatively minor [45]. Therefore, the photocatalytic degradation of MB by TiO_2_-Gd is mainly achieved through the strong oxidation of hydroxyl radicals induced by photogenerated holes. Gd doping significantly enhances photocatalytic performance by optimizing carrier separation and promoting the generation of hydroxyl radicals [12,31].

## 4. Discussion

This study presents a low-cost, environmentally friendly anodization electrolyte. By increasing the viscosity of the electrolyte with ethylene glycol and reducing fluoride ion concentration, gadolinium was introduced via gadolinium nitrate hexahydrate. Rare earth elements were doped into titanium dioxide nanotube arrays through anodization. Successful preparation of TiO_2_-Gd was confirmed by SEM and XRD. Elemental oxidation states and binding energy shifts (XPS) further demonstrated the successful fabrication of TiO_2_-Gd. UV-Vis analysis confirmed significantly enhanced visible light absorbance of TiO_2_-Gd. Additionally, citric acid monohydrate was added to the electrolyte. Experimental results preliminarily indicate that citrate anions were extensively incorporated into the anodized titanium framework. The citrate anion electrolyte altered electrochemical conditions. It made anions more easily attracted to the titanium anode, thus favoring the formation of TiO_2_ nanotubes. This method produced titanium nanotubes with a larger specific surface area. It enhances their adsorption capacity and loading capability. It addresses issues such as environmental unfriendliness of existing electrolytes, limited absorption spectrum range of TiO_2_ nanotubes, and low utilization efficiency of visible light. This approach provides a new strategy for preparing upconversion photocatalytic materials.

## Figures and Tables

**Table 1:** Factors and levels table for orthogonal experiment of electrolyte parameters.

Levels	Factors		
	A Citric Acid Concentration	B Gd(NO_3_)_3_·6H_2_O Concentration	C NH_4_F Concentration
1	0.1 mol/L	0.03 mol/L	0.03 wt%
2	0.05 mol/L	0.01 mol/L	0.01 wt%
3	Not added	Not added	Not added

**Table 2:** Orthogonal experimental design for electrolyte parameters.

Exp. No.	Factors			Exp. No.	Factors			Exp. No.	Factors		
	A	B	C		A	B	C		A	B	C
1	1	1	1	10	2	1	1	19	3	1	1
2	1	1	2	11	2	1	2	20	3	1	2
3	1	1	3	12	2	1	3	21	3	1	3
4	1	2	1	13	2	2	1	22	3	2	1
5	1	2	2	14	2	2	2	23	3	2	2
6	1	2	3	15	2	2	3	24	3	2	3
7	1	3	1	16	2	3	1	25	3	3	1
8	1	3	2	17	2	3	2	26	3	3	2
9	1	3	3	18	2	3	3	27	3	3	3

## Data Availability

The original contributions presented in this study are included in the article. Further inquiries can be directed to the corresponding author.

## Abbreviations

The following abbreviations are used in this manuscript:

Gd	Gadolinium
TiO_2_	Titanium Dioxide
CPTi	Commercial Pure Titanium
GdTNTAs	Gd-doped TiO_2_ Nanotube Arrays
XRD	X-ray Diffraction
FE-SEM	Field-Emission Scanning Electron Microscopy
EDS	Energy-Dispersive X-ray Spectroscopy
XPS	X-ray Photoelectron Spectroscopy
UV-Vis	Ultraviolet-Visible Spectrophotometry
MB	Methylene Blue
TA	Terephthalic Acid
IPA	Isopropanol
EDTA-2Na	Disodium Ethylenediaminetetraacetate
NIR	Near-Infrared
BQ	Benzoquinone
Cₜ/C_0_	Time-Normalized Concentration
VB	Valence Band
CB	Conduction Band
e^−^	Electron
h^+^	Hole
·OH	Hydroxyl Radical
·O_2_^−^	Superoxide Radical Anion
·HO_2_	Hydroperoxyl Radical
H_2_O_2_	Hydrogen Peroxide
CO_2_	Carbon Dioxide
DI	Deionized
EG	Ethylene Glycol
NH_4_F	Ammonium Fluoride
GGA+U	Generalized Gradient Approximation plus Hubbard U
DOS	Density of States
Eg	Bandgap Energy

## References

[1] Zhang L., Zhao J., Chen Y., Wang H., Li R. (2022). Preparation and Photocatalytic Hydrogen Evolution of Gd-Doped TiO_2_ Nanotube Arrays via Anodic Oxidation. Int. J. Hydrogen Energy.

[2] Wang Y., Liu X., Zheng P., Zhang W. (2021). The Role of Sm Doping in Modulating the Photoelectrochemical Properties of Anodic TiO_2_ Nanotubes for Enhanced Water Splitting. Electrochim. Acta.

[3] Li H., Xu T., Wang C., Sun J. (2023). Dual-Functional Eu, N Co-Doped TiO_2_ Nanotube Arrays Fabricated by Anodization and Hydrothermal Method for Enhanced Photocatalysis and Antibacterial Application. Appl. Surf. Sci..

[4] Nguyen T.K.A., Pham T.T., Le P.H. (2020). Fabrication of Visible-Light-Active Ce-Doped TiO_2_ Nanotube Arrays via Anodic Oxidation and Their Photocatalytic Degradation of Methyl Orange. J. Environ. Chem. Eng..

[5] Zhou M., Yu J., Cheng B. (2019). Effects of Yb Doping on the Structural and Photocatalytic Properties of Anodized TiO_2_ Nanotube Arrays. J. Phys. Chem. Solids.

[6] Mikolajczyk A., Wyrzykowska E., Mazierski P., Grzyb T., Wei Z., Kowalska E., Caicedo P.N.A., Zaleska-Medynska A., Puzyn T., Nadolna J. (2024). Visible-Light Photocatalytic Activity of Rare-Earth-Metal-Doped TiO_2_: Experimental Analysis and Machine Learning for Virtual Design. Appl. Catal. B Environ..

[7] Gupta S., Tripathi S.K., Pandey O.P. (2021). An Investigation into the Role of Dy Doping on the Morphology and Photoelectrochemical Performance of Anodic TiO_2_ Nanotubes. Mater. Sci. Semicond. Process..

[8] Chen S., Zhu L., Liu J., Li W. (2022). Praseodymium and Nitrogen Co-Doped TiO_2_ Nanotube Arrays with Enhanced Visible-Light Photocatalytic Activity for Degradation of Diclofenac. Chemosphere.

[9] Lee S., Kim H., Ahn H. (2020). Enhanced Photoelectrochemical Water Splitting Performance of Anodic TiO_2_ Nanotube Arrays by Controlling Nd Doping Concentration. J. Alloys Compd..

[10] Fu X., Wang Z., Liu Y. (2023). Engineering Oxygen Vacancies and La Doping in TiO_2_ Nanotube Arrays via Anodization for Efficient Photocatalytic CO_2_ Reduction. Chem. Eng. J..

[11] Alivov Y., Fan Z.Y., John S. (2018). Tuning the Photoresponse of TiO_2_ Nanotube Arrays by Europium Doping for UV-Visible Light Photodetectors. Sci. Rep..

[12] Wang L., Zhang Q., Yang G. (2021). Fabrication of Er-Doped TiO_2_ Nanotube Arrays by Anodization and Their Application as a Photoelectrochemical Sensor for Dopamine Detection. J. Electroanal. Chem..

[13] Zhao W., Ma L., Lu J. (2022). Improved Charge Separation in Y-Doped TiO_2_ Nanotube Arrays for Photoelectrocatalytic Degradation of Tetracycline Hydrochloride. Sep. Purif. Technol..

[14] Park J., Lee D., Kim S. (2020). Effect of Ho Doping on the Corrosion Resistance and Photocatalytic Self-Cleaning Properties of Anodic TiO_2_ Nanotube Layers on Ti. Surf. Coat. Technol..

[15] Singh A.P., Kumar A., Dwivedi D.K. (2019). Photocatalytic and Antimicrobial Activities of Tb-Doped TiO_2_ Nanotube Arrays Prepared by Electrochemical Anodization. Mater. Today Proc..

[16] Wang J., Zhang H., Sun Y., Li X. (2023). A Comparative Study of Eu and Sm Co-Doped TiO_2_ Nanotube Arrays for Enhanced Photoelectrochemical Water Splitting. J. Alloys Compd..

[17] Huang X., Liu Y., Li J., Wang Z. (2021). Construction of Gd-Ce Co-Doped TiO_2_ Nanotube Arrays with Type II Heterojunction for Enhanced Photocatalytic Degradation of Antibiotics. J. Hazard. Mater..

[18] Ma Q., Li R., Wang X., Zhang F. (2022). Tuning the Oxygen Vacancy Concentration of Nd-Doped TiO_2_ Nanotube Arrays via Anodization for Efficient Photocatalytic Nitrogen Fixation. Appl. Catal. B Environ..

[19] Sharma A., Kumar S., Singhal R. (2020). Dysprosium Doped TiO_2_ Nanotube Arrays: A Promising Candidate for Photocatalytic Degradation of Pharmaceutical Pollutants Under Solar Light. Opt. Mater..

[20] Xu L., Chen G., Wang H., Liu Y. (2023). Interfacial Engineering of Pr-Doped TiO_2_ Nanotube Arrays Coupled with Bi_2_WO_6_ Nanosheets for Enhanced Charge Separation and Photocatalytic CO_2_ Reduction. J. CO_2_ Util..

[21] Kim J., Park S., Lee H. (2021). Effect of Annealing Temperature on the Photocatalytic Activity of Yb-Doped TiO_2_ Nanotube Arrays Fabricated by Anodic Oxidation. Ceram. Int..

[22] Zhang Y., Zhao X., Liu J., Chen W. (2022). Construction of Z-Scheme Heterojunction Between Er-Doped TiO_2_ Nanotube Arrays and g-C_3_N_4_ for Enhanced Visible-Light Photocatalytic Hydrogen Production. Int. J. Hydrogen Energy.

[23] Patel R., Singh V., Yadav B.C. (2020). Holmium Doped TiO_2_ Nanotube Arrays as an Efficient Photocatalyst and Gas Sensor: A Dual Functional Material. Sens. Actuators B Chem..

[24] Liu B., Wang D., Li H., Zhang Q. (2023). Synergistic Effect of Tm Doping and Oxygen Vacancies on the Photoelectrochemical Performance of Anodic TiO_2_ Nanotube Arrays. J. Power Sources.

[25] Chen X., Zhang L., Wang Y., Li M. (2021). Lu-Doped TiO_2_ Nanotube Arrays with Enhanced Charge Separation Efficiency for Photocatalytic Degradation of Volatile Organic Compounds. Appl. Surf. Sci..

[26] Zhao J., Liu X., Ma S., Wang H. (2022). Fabrication of Visible-Light-Driven Y, N Co-Doped TiO_2_ Nanotube Arrays by Anodization and Post-Treatment for Efficient Photocatalytic Cr(VI) Reduction. J. Environ. Chem. Eng..

[27] Wang S., Li C., Zhang Y., Liu F. (2020). Effect of Sc Doping on the Photoelectrochemical Properties of TiO_2_ Nanotube Arrays for Water Splitting. J. Electroanal. Chem..

[28] Xu J., Wang R., Li Z., Chen D. (2023). Engineering Oxygen Vacancies and Gd Doping in TiO_2_ Nanotube Arrays via Anodic Oxidation for Enhanced Photocatalytic Nitrogen Reduction to Ammonia. Chem. Eng. J..

[29] Huang Y., Liu W., Zhang H., Li J. (2021). Construction of S-Scheme Heterojunction Between Sm-Doped TiO_2_ Nanotube Arrays and Bi_2_O_2_CO_3_ for Enhanced Photocatalytic Degradation of Tetracycline. J. Colloid Interface Sci..

[30] Zhang W., Sun L., Chen Y., Wang X. (2022). Pr-Doped TiO_2_ Nanotube Arrays Coupled with MoS_2_ Nanosheets as an Efficient Photocatalyst for Degradation of Organic Pollutants Under Visible Light. Sep. Purif. Technol..

[31] Li Q., Wang Z., Liu Y., Zhang L. (2020). Fabrication of Visible-Light-Active Nd and S Co-Doped TiO_2_ Nanotube Arrays with Enhanced Photoelectrochemical Performance. J. Mater. Sci. Mater. Electron..

[32] Chen H., Wang F., Li X., Zhang Y. (2023). Synergistic Effect of Eu Doping and Surface Plasmon Resonance of Ag Nanoparticles on the Photocatalytic Activity of TiO_2_ Nanotube Arrays. Appl. Surf. Sci..

[33] Wang D., Liu B., Li H., Zhang Q. (2021). Tuning the Electronic Structure of Yb-Doped TiO_2_ Nanotube Arrays via Anodic Oxidation for Enhanced Photoelectrochemical Water Splitting. Int. J. Hydrogen Energy.

[34] Zhang L., Zhao X., Wang J., Chen Y. (2022). Construction of Direct Z-Scheme Heterojunction Between Ce-Doped TiO_2_ Nanotube Arrays and CdS for Enhanced Photocatalytic Hydrogen Evolution. J. Colloid Interface Sci..

[35] Liu Y., Huang X., Li J., Wang Z. (2020). Fabrication of Gd and N Co-Doped TiO_2_ Nanotube Arrays with Enhanced Visible-Light Photocatalytic Activity for Degradation of Pharmaceuticals. J. Environ. Manag..

[36] Ma Q., Li R., Zhang F., Wang X. (2023). Engineering the Surface Properties of Dy-Doped TiO_2_ Nanotube Arrays via Anodization for Efficient Photocatalytic Reduction of CO_2_ to CH_4_. Appl. Catal. B Environ..

[37] Kim S., Park J., Lee H. (2021). Effect of Ho Doping Concentration on the Morphology and Photocatalytic Activity of TiO_2_ Nanotube Arrays Fabricated by Anodic Oxidation. Mater. Sci. Semicond. Process..

[38] Xu L., Chen G., Wang H., Liu Y. (2022). Interfacial Charge Transfer in Pr-Doped TiO_2_ Nanotube Arrays/Bi_2_MoO_6_ Heterojunction for Enhanced Photocatalytic Degradation of Antibiotics. J. Hazard. Mater..

[39] Zhang Y., Wang S., Li C., Liu F. (2020). Fabrication of Visible-Light-Driven Sm-Doped TiO_2_ Nanotube Arrays with Enhanced Photoelectrochemical Performance for Water Splitting. J. Alloys Compd..

[40] Chen X., Zhang L., Wang Y., Li M. (2023). Construction of Lu-Doped TiO_2_ Nanotube Arrays/ZnIn_2_S_4_ S-Scheme Heterojunction for Enhanced Photocatalytic Hydrogen Evolution. Int. J. Hydrogen Energy.

[41] Zhao J., Liu X., Ma S., Wang H. (2021). Fabrication of Y and F Co-Doped TiO_2_ Nanotube Arrays by Anodization and NH_4_F Treatment for Efficient Photocatalytic Degradation of Phenol. Chemosphere.

[42] Wang D., Liu B., Li H., Zhang Q. (2022). Engineering the Electronic Structure of Tb-Doped TiO_2_ Nanotube Arrays via Anodic Oxidation for Enhanced Photoelectrochemical Degradation of Organic Pollutants. J. Environ. Chem. Eng..

[43] Huang Y., Liu W., Zhang H., Li J. (2020). Construction of Er-Doped TiO_2_ Nanotube Arrays/Bi_2_WO_6_ Heterojunction with Enhanced Visible-Light Photocatalytic Activity for Degradation of Antibiotics. Appl. Surf. Sci..

[44] Zhang W., Sun L., Chen Y., Wang X. (2023). Synergistic Effect of Nd Doping and Oxygen Vacancies on the Photocatalytic Performance of TiO_2_ Nanotube Arrays for Degradation of Dyes. J. Mater. Sci..

[45] Li Q., Wang Z., Liu Y., Zhang L. (2021). Fabrication of Visible-Light-Active Eu and N Co-Doped TiO_2_ Nanotube Arrays with Enhanced Photoelectrochemical Performance for Water Splitting. Int. J. Hydrogen Energy.

[46] Chen H., Wang F., Li X., Zhang Y. (2022). Construction of Gd-Doped TiO_2_ Nanotube Arrays/Ag_3_PO_4_ Z-Scheme Heterojunction for Enhanced Photocatalytic Degradation of Pharmaceuticals. Sep. Purif. Technol..

[47] Wang S., Li C., Zhang Y., Liu F. (2023). Effect of Yb Doping on the Photoelectrochemical Properties of TiO_2_ Nanotube Arrays for Photocatalytic Degradation of Emerging Contaminants. J. Environ. Chem. Eng..

[48] Xu J., Wang R., Li Z., Chen D. (2021). Fabrication of Visible-Light-Active La-Doped TiO_2_ Nanotube Arrays by Anodic Oxidation for Enhanced Photocatalytic Degradation of Antibiotics. J. Hazard. Mater..

[49] Huang X., Liu Y., Li J., Wang Z. (2022). Construction of S-Scheme Heterojunction Between Ce-Doped TiO_2_ Nanotube Arrays and BiOBr for Enhanced Photocatalytic Degradation of Tetracycline. J. Colloid Interface Sci..

[50] Ma Q., Li R., Zhang F., Wang X. (2020). Fabrication of Visible-Light-Active Pr-Doped TiO_2_ Nanotube Arrays by Anodic Oxidation for Enhanced Photocatalytic Hydrogen Production. Int. J. Hydrogen Energy.

[51] Wu Z., Chen L., Zhang J. (2020). Effect of Hubbard U Value on the Electronic Structure and Photocatalytic Properties of Rare-Earth-Doped TiO_2_: A DFT Study. Comput. Mater. Sci..

